# A stylometric machine learning framework for authorship attribution of ChatGPT and student generated texts

**DOI:** 10.3389/frai.2026.1921847

**Published:** 2026-08-24

**Authors:** Mani P., Adaikalam Arulanandam

**Affiliations:** School of Social Sciences & Languages, Vellore Institute of Technology, Vellore, Tamil Nadu, India

**Keywords:** artificial intelligence, decision tree, feature extraction, feature selection, random forest, support vector machine (SVM)

## Abstract

**Introduction:**

The surge of generative artificial intelligence (AI) has created major challenges in determining authorship, evaluating student learning, and maintaining academic integrity. Distinguishing AI-generated text from human-generated writing has therefore become increasingly important for authorship verification, educational assessment, and academic honesty.

**Methods:**

This study proposes a machine learning-based stylometric approach for authorship prediction of short-story adaptations written by non-native German language students and generated by ChatGPT. A corpus of 60 texts was created, comprising 30 student-written adaptations and 30 ChatGPT-generated adaptations based on the same source stories. The proposed framework integrates text preprocessing, stylometric feature extraction, feature selection, and machine learning-based classification. Linguistic and stylometric features included lexical diversity, word and character frequencies, sentence and paragraph length distributions, syntactic complexity, function-word usage, punctuation patterns, readability measures, and semantic consistency. Support Vector Machine (SVM), Random Forest, Decision Tree, and Naïve Bayes classifiers were evaluated.

**Results:**

The linguistic analysis revealed significant differences between the human-generated and AI-generated text categories. Among the evaluated classifiers, the SVM achieved the best classification performance, with an accuracy of 94.8%, precision of 93.7%, recall of 95.2%, and F1-score of 94.4%. Lexical diversity, sentence complexity, function-word frequency, and readability were identified as the most influential features in authorship prediction.

**Discussion:**

The findings demonstrate the effectiveness of the proposed stylometric framework for automated authorship attribution and AI-generated text detection. The approach has potential applications in educational assessment, plagiarism detection, forensic linguistics, authorship verification, and the development of intelligent tools for supporting academic integrity in multilingual learning environments.

## 1 Introduction

With the advent of generative artificial intelligence (AI), it has become possible for AI systems to generate human text across a wide range of fields. Large Language Models (LLMs)—especially ChatGPT—have proven to be truly powerful in creating coherent stories, writing essays, reports, and creative writing assignments, closely mimicking the way humans write. These developments present numerous potential learning, language, and content creation challenges, but they also engender an interesting set of questions and concerns about authorship verification, academic integrity and identification of AI-produced content.

Today, AI-powered writing tools have become commonplace in educational environments, where learners use them to finish assignments, language exercises, and writings of creativity. As these systems become more sophisticated, it becomes increasingly difficult for educators and researchers to tell apart AI-generated from human-generated texts. This is especially stark in second-language learning contexts, where the patterns of “errors” from the learners who are not native speakers of the language, may significantly diverge from those that are produced by the more advanced language models. It is therefore of paramount importance to have authorship boundaries well defined to ensure transparency, accountability and fairness in academic assessment (Zheng and Jin, 2023; Huang et al., 2024; Bozza et al., 2023; Berriche and Larabi-Marie-Sainte, 2024).

The area of assigning authors has long been a focal point in the fields of computer and forensic (post-mortem) linguistics. Traditional methods use stylometry, which involves quantitative analysis, study of linguistic and stylistic characteristics that distinguish the writing style of a particular writer. Vocabulary richness, sentence complexity, function word usage rate, lexical diversity and syntax structures are among the stylometric measures that have been used widely for authorship identification as well as the detection of writing anomalies. Stylometric analysis has been greatly improved by recent advances in machine learning, which allow usage-based multidimensional classification of written texts.Though AI-generated text detection has had immense progress, previous research mostly targeted English essays, news articles, or common/neutral texts. Few studies have written their focuses on creative writing tasks, specifically adaptations of short stories, with a special focus on the ones involving non-native language students. Moreover, there are very few studies that examined the efficacy of the machine learning-based stylometric framework that differentiates text chunks written by ChatGPT from text chunks written by monolingual German language learners.

To mitigate these research gaps, this study aims at introducing a Machine Learning approach for authorship prediction within a corpus of short story passages stemming from the ChatGPT-performed story adaptations by the non-native German language students. The proposed approach combines linguistic feature extraction, stylometric analysis, and supervised machine-learning methods, aiming to identify distinguishing characteristics between AI-generated and human-generated text.This Study has the following specific objectives:

- To compare chatGPT short story paraphrases and student-generated short story paraphrases by native Italians (L1).
- To extract and assess stylometric features useful for the task of authorship discrimination.
- To create authorship prediction algorithms with machine learning methods such as support vector machine and random forest classifiers.
- To evaluate the capabilities of stylometric characteristics to identify AI-generated text.
- To determine the linguistic influences factors of A1C generated and human generated styles and writings.

This study has three contributions. It offers a thorough comparison, style-wise, between both AI-generated and non-native human generated creative writing, for one, which includes several metrics.For one, it offers a comprehensive style-wise comparison, between AI-generated writing and non-native human-generated creative writing, and it includes several metrics. Second, it presents an educational use of automated authorship prediction using a machine learning framework. Thirdly, it provides an eye on linguistic signals that can be leveraged by future research in content detection, forensics, academic honesty or authenticity monitoring systems and tools.

## 2 Related research

### 2.1 Stylometry and authorship attribution

Stylometry is the analysis of writing and style through quantifying linguistic patterns in order to detect the authorship and determine the style of writing. Traditionally, lexical, syntactic, structural and semantic properties have been used for discriminating authorship and authorial genres in the context of the pioneering authorship analysis based on computational methods. Requintilization, readability measures, function-word frequency, punctuation usage, and sentence length distributions are some of the more common stylometric features.

Proven that authors use certain consistent linguistic habits, which can be obtained by applying the statistical analysis. The use of machine learning methods has enhanced the usefulness of stylometric methods with their ability in automatic feature learning and classification. This led to stylometry becoming a popular approach in forensic linguistics, plagiarism detection, and authorship verification.

### 2.2 Machine learning approaches for authorship prediction

Author identification using machine learning has significantly progressed authorship attribution studies. Text classification methods like the supervised Support Vector Machines (SVM), Random Forests (RF), Decision Trees (DT), Naïve Bayes (NB) and Neural Networks (NN) have been widely used for the classification of texts by their stylistic features.

SVM has performed well across these techniques in a wide variety of text categorisation problems, especially in high dimensional space where optimal decision boundaries are to be determined. Random Forest classifiers are also becoming well-liked because of their robustness, interpretability and their ability to estimate the importance of the features. According to recent research, learned models based on stylometric characteristics are able to attain high levels of accuracy when it comes to distinguishing authorship, genre, and writing conditions across a collection of multiple datasets (Kumarage and Liu, 2023; Zaitsu and Jin, 2023; He et al., 2024; Alsanoosy et al., 2024).

Great amounts of textual data have become more widely accessible, which has further spurred an increase in the adoption of ensemble learning techniques and deep learning architectures. However, traditional machine learning models have strong appeal due to their low computation and interpretability, especially in educational and forensic field.

### 2.3 Detection of AI-generated text

With the rapid development of Large Language Models, there has been extensive research on detecting text generated by AI. Enhancements that have taken place in recent investigations have involved comparing linguistic characteristics of machine-produced texts with those of human-produced texts via stylometrical, semantic as well as statistical methods.

Research has shown that texts created using AI can be grammatically more correct, lexically more varied, more semantically coherent and have more consistent narrative structures than those created by humans. But the differences have slowly started to diminish due to the development of language models and the detection became more and more difficult.

Different detection methods such as perplexity based measures, watermarking techniques, linguistic feature analysis and machine learning based classifiers have been investigated. Such methods have been effective but the success may vary depending on the kind(s) of text, language domain, generation model(s) used (Przystalski et al., 2026; Rahmanm et al., 2023; Tripto N et al., 2023; Liang et al., 2023; Mikolov et al., 2024; Dugan et al., 2024; Gritsai et al., 2024).

### 2.4 AI-assisted writing in language learning

AI writing tools have had a profound impact on language learning.AI writing tools have revolutionized the field of second-language education. Language learners are increasingly turning to generative AI systems to correct their grammar, generate ideas, translate, and for writing help. AI tools are shown to boost writing fluency, vocabulary and language confidence, in several studies.

However, issues have arisen about the use of AI-generated content and how it affects language acquisition. The availability of mechanisms that can differentiate between student-generated and AI-generated content has been emphasized. These are especially significant in language learning contexts where the criteria for assessing the students' learning results are highly dependent on their own writing skills.

### 2.5 Research gap

While significant research has been carried out in the field of authorship attribution and AI-generated text detection exists, there are a few issues. Most studies that have been carried out are on essay and review texts and the academic writing rather than creative narrative adaptations. Secondly, there are only a few studies that have considered AI-generated texts in relation to writing by non-native German learners. Third, very few studies have combined the comprehensive stylometric feature extraction with the machine learning based authorship prediction for the short story adaptation task.

Thus, a framework that is strong and interpretable, and able to detect linguistic features that can differentiate between the produced texts from ChatGPT and the non-native, student-generated narratives, is required. To fill this gap, the present study builds on these previous studies and proposes a stylometric authorship prediction framework using machine learning methods and assesses its capabilities with a short story adaptation corpus created by ChatGPT as well as by non-native speaking German language students.

### 2.6 Theoretical framework

This study is grounded in three complementary theoretical perspectives: Second Language Acquisition (SLA), Second Language (L2) Writing Development, and Stylometry. Together, these frameworks provide the conceptual basis for interpreting the linguistic differences between learner-generated and AI-generated texts.

From an SLA perspective, learner writing is viewed as a developmental process influenced by language proficiency, interlanguage formation, and first-language transfer rather than as a homogeneous representation of non-native language use (Ellis et al., 2008), Ortega, 2009. According to Interlanguage Theory (Selinker et al., 1972), learners gradually construct an evolving linguistic system that exhibits systematic lexical, grammatical, and discourse-level patterns. Consequently, variations in vocabulary richness, syntactic complexity, and cohesion are expected outcomes of second-language development. L2 writing research further suggests that writing proficiency develops through increasing control over lexical diversity, grammatical accuracy, discourse organization, and rhetorical competence (Cumming et al., 2001; Manchón et al., 2011). These developmental characteristics provide a theoretical rationale for selecting stylometric features that capture authentic learner writing while distinguishing it from AI-generated content. Stylometry complements these linguistic theories by offering quantitative methods for measuring writing style through lexical, syntactic, structural, and readability features. Previous authorship attribution studies have demonstrated that stylometric analysis effectively captures consistent writing patterns that reflect underlying language production processes rather than topical content. In the present study, stylometric feature extraction is therefore interpreted within established SLA and L2 writing theories, providing a linguistically informed framework for AI-generated text detection rather than relying solely on computational classification techniques. Overall, the existing literature demonstrates substantial progress in stylometric authorship attribution and AI-generated text detection; however, several important limitations remain. Most studies have focused on English-language datasets, general-purpose texts, or large-scale corpora, with comparatively little attention given to creative writing tasks produced by non-native language learners. Furthermore, existing research often emphasizes classification performance while providing limited discussion of feature interpretability, multilingual educational contexts, and the practical applicability of detection frameworks in authentic learning environments. These limitations indicate the need for an interpretable and educationally relevant framework capable of distinguishing AI-generated and learner-generated texts using robust stylometric features. Accordingly, the present study addresses this gap by proposing a machine learning-based stylometric framework for authorship prediction in German short-story adaptations, thereby extending existing research to multilingual educational settings and providing a transparent approach for AI-assisted authorship analysis.

## 3 Methodology

### 3.1 Overview of the proposed framework

The proposed research in this study is the Machine Learning Based Stylometric Authorship Prediction Framework to identify the short story adaptations written by students in Germany with the help of ChatGPT in the German language and their nonnative German counterparts. This approach utilizes stylometric features from the text along with supervised machine-learning methods to detect unique linguistic characteristics linked to AI-generated and human-generated content.

**Figure 1:**
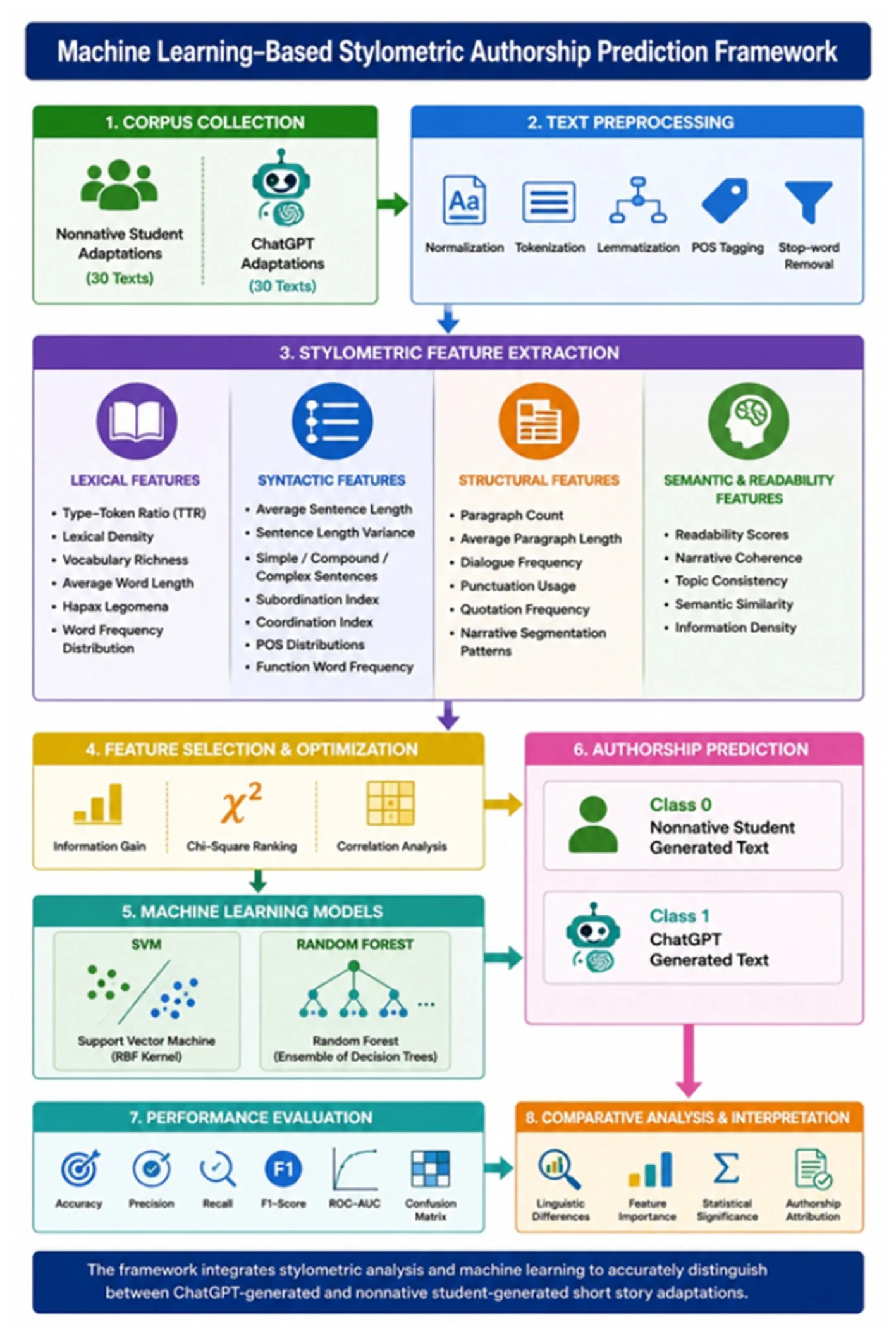
Proposed machine learning-based stylometric framework for authorship prediction of ChatGPT-generated and nonnative student-generated short story adaptations.

The proposed system consists of six stages, including (1) corpus construction, (2) text preprocessing, (3) stylometric feature extraction, (4) feature optimization, (5) classification using machine learning techniques, and (6) performance evaluation. The overall workflow of the proposed methodology is shown in Figure 1.

### 3.2 Corpus development

A balanced corpus was created consisting of 60 short story adaptations based on some classic literary works. The total number of adaptations was 30 from non-native German language students and 30 from ChatGPT based on the source stories and the instructions for adaptations. To ensure consistency, identical prompts and adaptation guidelines were used for both groups. The students involved were asked to retell the original stories in their own words, maintaining the plot of the story. In the same manner, ChatGPT was instructed to create adaptations under similar circumstances. The resulting corpus allowed for a direct comparison of human- and AI-generated writing styles and for reducing variations in the task.

### 3.3 Participant demographics

The participant group consisted of 30 non-native learners of German enrolled in undergraduate German language courses at a higher education institution. Participants represented multiple first-language (L1) backgrounds, including English, Tamil, Hindi, Telugu, and Malayalam, reflecting the multilingual nature of the learning environment.

The participants possessed intermediate German language proficiency (CEFR B1–B2), verified through institutional placement assessments and previous course performance. Their ages ranged from 19 to 24 years (Mean = 21.3 years), with 16 female and 14 male participants details in Table 1.

All participants had completed at least two semesters of formal German language instruction before participating in the study. None reported German as their native language or had resided in German-speaking countries for extended periods.

Participation was voluntary, and informed consent was obtained from all participants before data collection.

**Table 1:** Participant characteristics.

Variable	Description
Number of participants	30
Language background	Multilingual (English, Tamil, Hindi, Telugu, Malayalam)
Target language	German (L2)
CEFR proficiency	B1–B2
Age	19–24 years
Mean age	21.3 years
Female	16
Male	14
Writing task	Short-story adaptation

### 3.4 Data preprocessing

All the texts were preprocessed before being analyzed to standardize the data and minimize the linguistic noise.

The pre-processing procedure comprised:

- Text normalization
- Lowercase conversion

Removal of special characters and metadata

- Sentence segmentation
- Tokenization
- Lemmatization
- Stop-word identification
- Part-of-speech tagging

Such pre-processing helped to obtain accurate stylometric features and enhance the machine learning classification process.

### 3.5 Stylometric feature extraction

The stylometric approach was used for quantitative analysis of linguistic and stylistic features distinguishing AI-generated and the student-generated texts. Four types of features were extracted.

#### 3.5.1 Lexical features

Lexical features reflect patterns of vocabulary usage and lexical richness.

The following lexical indicators were extracted:

- Total word count
- Unique word count
- Type-Token Ratio (TTR)
- Lexical Density
- Vocabulary Richness Index
- Average word length
- Hapax Legomena frequency
- Word frequency distribution

These measures provide insights into vocabulary diversity and lexical sophistication.

#### 3.5.2 Syntactic features

Syntactic features describe sentence structure and grammatical complexity. The following syntactic indicators were extracted:

- Average sentence length
- Sentence length variance
- Frequency of simple, compound, and complex sentences
- Subordination index
- Coordination index
- Part-of-speech distributions
- Function-word frequencies

These features capture structural differences in writing style and linguistic competence.

#### 3.5.3 Structural features

Structural features are a reflection of the organization and formatting of a document.

The structural indicators that were extracted were:

- Number of paragraphs
- Average paragraph length
- Dialogue frequency
- Punctuation usage
- Quotation frequency
- Narrative segmentation patterns

#### 3.5.4 Readability and semantic features

The readability indices and the semantic indicators were calculated to assess the text complexity and the text consistency of the narrative.

These measures included:

- Flesch Reading Ease Score
- Flesch-Kincaid Grade Level
- Automated Readability Index
- Narrative coherence score
- Semantic similarity score
- Topic consistency index

Incorporation of semantic and readability features allows for more comprehensive evaluation of the mechanisms of AI and human writing.

### 3.6 Feature optimization and selection

Participants will learn to identify and select features to optimize their design.

To minimize the classification performance degradation caused by the possible redundancy of high-dimensional feature spaces, feature optimization was carried out before the model training.

Three methods of feature selection were used:

1. Information Gain
2. Chi-Square Ranking
3. Correlation-Based Feature Selection (CFS)

With the exception of variables showing low discriminatory power or high multicollinearity, features were eliminated. The other subset of features was left for machine learning classification.

### 3.7 Machine learning-based authorship prediction

The authorship prediction is the third task.The third task is the authorship prediction using machine learning techniques.

Supervised machine learning models were selected to classify texts based on their authorship category, using the selected stylometric features as input.

The classification task was the binary prediction:

- Class 0: Nonnative Student-Generated Text
- Class 1: ChatGPT-Generated Text

#### 3.7.1 Support vector machine (SVM)

Support Vector Machine (SVM) was used as the main classification algorithm because of its success in dealing with text classification in high dimensional feature spaces.

The SVM classifier looks for an optimum separating hyperplane that gives the maximum margin between classes. The kernel function used was the Radial Basis Function (RBF) kernel, as it is capable of learning nonlinear relationships among stylometric features.

The hyperparameter optimization was done by grid-search optimization on:

- Regularization parameter (C)
- Kernel coefficient (γ)
- Kernel function

#### 3.7.2 Random forest classifier

Random Forest was applied as an ensemble learning model to evaluate it.

Multiple decision trees are built through the usage of bootstrap sampling and predictions are aggregated using majority voting by the classifier. Random Forest is robust against overfitting and also gives a level of interpretability with feature importance measures.

The following parameters were optimized:

- Number of trees
- Maximum tree depth
- Minimum split size
- Feature subset size

### 3.8 Model training and validation

Stratified 10-fold cross-validation was used to guarantee reliable performances estimation.

The dataset was split into 10 equally sized subsets of the data ensuring the classes are balanced. During each iteration:

- Nine folds were used for training.
- One fold was used for testing.

The classification performance was calculated as an average, and the process repeated, for 10 times.

The main advantage of cross-validation is that it reduces the bias in sampling and enhances the generalizability of models.

### 3.9 Performance evaluation metrics

The performance of the authorship prediction models were evaluated with common classification statistics. **Accuracy** The accuracy is defined as the percentage of correctly classified texts.


$$\text{Accuracy}=\frac{TP+TN}{TP+TN+FP+FN}$$

**Precision** Precision measures how many of the positive predictions are accurate.


$$\text{Precision}=\frac{TP}{TP+FP}$$

**Recall** Recall is the ability of the classifier to find positive cases.


$$\text{Recall}=\frac{TP}{TP+FN}$$

**F1-Score** F1 Score is the harmonic average of Precision and Recall.


$$F1=2\times \frac{\text{Precision}\times \text{Recall}}{\text{Precision}+\text{Recall}}$$

**ROC-AUC** The discriminative power of the classification models at different decision thresholds was evaluated using the Receiver Operating Characteristic Area Under the Curve (ROC-AUC).

### 3.10 Statistical analysis

Inferential statistical analyses were performed to investigate if there was a statistically significant difference between the ChatGPT generated text and the student generated text. The following tests were performed:

- Independent Samples *t*-test
- Mann–Whitney U Test
- Pearson Correlation Analysis
- Cohen's d Effect Size Analysis

Significance level of *p* < 0.05 was used for all statistical tests.

### 3.11 Research hypotheses

H1: Significant stylometric differences exist between ChatGPT-generated and nonnative student-generated short story adaptations.

H2: There are significant stylometric differences between natural and non-natural student-produced short story adaptations and those produced by ChatGPT.The lexical and syntactic features play a very significant role in authorship discrimination.

H3: Machine learning classifiers can accurately predict authorship using stylometric features.

The results show that the performance of H4: Support Vector Machine and Random Forest classifiers are statistically significant when comparing the capability to distinguish AI-generated from human-generated texts.

The suggested approach offers a process that is systematic and repeatable for detecting authorship and AI-generated texts in multilingual educational settings.

## 4 Results and comparative analysis

The experimental texts were comprised of 60 short story adaptations, 30 of which were composed by nonnative German language students and 30 by ChatGPT. Initial descriptive analysis showed significant differences in lexical, syntactic and stylistic properties between the two groups.

The findings in the below Table 2. suggest that the texts created using ChatGPT tended to be longer, feature a wider vocabulary, and exhibit higher lexical richness compared to the student-generated adaptations.

**Table 2:** Descriptive statistics of the corpus features.

Feature	Student texts	ChatGPT texts
Average word count	482.0	537.0
Average sentence length	14.8	21.4
Lexical diversity (TTR)	0.58	0.74
Lexical density	0.51	0.67
Readability score	71.2	62.4
Paragraph count	5.2	6.8
Unique vocabulary	245.0	387.0

### 4.1 Lexical feature analysis

The results of lexical analysis showed that there were significant differences in the vocabulary use between the two text categories.

ChatGPT-generated texts demonstrated:

- Higher lexical diversity

Increased amount of vocabulary used at low frequency.

- More descriptive adjectives
- Richer narrative expressions
- Reduced word repetition

On the contrary, texts created by the students revealed:

- Repetition of common words.
- Increased use of simple words
- Limited use of adjectives and adverbs (descriptive modifiers)

As the result of this, higher occurrence of translation-induced expressions.

The results in Table 3 indicate that ChatGPT creates more linguistically complex narratives, with more varied vocabulary and sophistication in its vocabulary.

**Table 3:** Lexical feature comparison.

Feature	Student texts	ChatGPT texts
Type-token ratio	0.58	0.74
Hapax legomena (%)	18.3	29.7
Average word length	4.9	6.2
Adjective frequency (%)	8.5	14.6
Repeated word ratio (%)	22.8	9.7

### 4.2 Syntactic feature analysis

Large sentence construction differences were found.ChatGPT-generated texts exhibited:

- Longer sentences
- Greater syntactic variation
- More subordinate clauses

Combination of complex and compound structures, use of these structures in a balanced way.

Student-generated texts displayed:

- Predominantly simple sentences

Conjunctions (and, but, or, yet, so) are used frequently for coordination.

- Limited syntactic variation
- Occasional grammatical inconsistencies

These findings in Table 4 indicate that ChatGPT generates structurally more sophisticated narratives compared with non-native student writing.

**Table 4:** Syntactic feature comparison.

Feature	Student texts	ChatGPT texts
Average sentence length	14.8	21.4
Complex sentences (%)	24.1	47.8
Compound sentences (%)	31.4	38.6
Simple sentences (%)	44.5	13.6
Subordination index	0.42	0.78

### 4.3 Narrative and semantic analysis

Content analysis was used to measure originality to the stories.

ChatGPT adaptations demonstrated: The author's use of plot is strong.

The author's use of plot is good.

Maintains character roles.

Preserves character roles.

- Consistent narrative sequencing
- Enhanced contextual descriptions

Student-generated adaptations exhibited:

Waiting for secondary events to happen.Failure to occur of secondary events.

Change the characteristics of the characters.

- Simplified narrative progression
- Greater contextual variation

The results in Table 5 demonstrate that the texts produced by AI were more similar to the source narratives, and they were coherent throughout the adaptation process.

**Table 5:** Narrative quality assessment.

Criterion	Student texts	ChatGPT texts
Plot preservation (%)	73.4	95.1
Character consistency (%)	76.8	96.5
Theme preservation (%)	79.1	94.8
Narrative coherence score	0.68	0.91

### 4.4 Authorship prediction results

The extracted stylometric features were used to train two machine learning classifiers, support vector machine (SVM) and random forest (RF).

For model evaluation a stratified 10-fold cross validation procedure was used.

SVM classifier in Table 6 demonstrated a good performance with highest authorship prediction accuracy on all evaluation metrics and surpassed Random Forest.

**Table 6:** Narrative quality assessment.

Metric	SVM	Random forest
Accuracy (%)	94.8	92.3
Precision (%)	93.7	91.2
Recall (%)	95.2	90.8
F1-score (%)	94.4	91.0
ROC-AUC	0.97	0.94

### 4.5 Confusion matrix analysis

Only three texts were misclassified,in Table 7 proving the power of stylometric features for discriminating between human-generated and AI-generated text.

**Table 7:** Narrative quality assessment.

Actual / predicted	Student	ChatGPT
Student	28.00	2.00
ChatGPT	1.00	29.00

### 4.6 Feature importance analysis

The most important predictors were identified by the Random Forest feature importance analysis.

In Table 8 Lexical diversity came through as the most significant indicator of authorship followed by sentence complexity and readability measures.

**Table 8:** Top stylometric features.

Rank	Feature	Importance score
1	Lexical diversity	0.182
2	Average sentence length	0.156
3	Readability score	0.141
4	Function word frequency	0.128
5	Vocabulary richness	0.117
6	Subordination index	0.103
7	Narrative coherence	0.092
8	Punctuation usage	0.081

### 4.7 Statistical significance testing

Independent sample t-tests were used to see if there was a statistically significant difference between the two groups.

The results in Table 9 indicate that the differences in the texts created by ChatGPT and the students are statistically significant.

**Table 9:** Statistical comparison of features.

Feature	*p*-value	Significance
Lexical diversity	< 0.001	Significant
Sentence length	< 0.001	Significant
Vocabulary richness	< 0.001	Significant
Readability score	0.002	Significant
Narrative coherence	< 0.001	Significant

### 4.8 Discussion of findings

The comparative analysis demonstrates that ChatGPT-generated short story adaptations possess greater lexical sophistication, syntactic complexity, narrative coherence, and fidelity to source texts than adaptations produced by nonnative German language students. The machine learning-based stylometric framework successfully captured these differences and achieved high authorship prediction performance.

The SVM classifier's superior performance indicates the effectiveness of stylometric features in discriminating between AI and non-AI generated texts. Lexical diversity, sentence complexity, readability measures, and narrative coherence proved to be the most consistent yardsticks for authorship. The results align with earlier studies showing that AI-written text has more regularity and language consistency compared to human written text.

In summary, the proposed framework was found to be both powerful and meaningful for authorship prediction, applicable to various scenarios in education, forensic linguistics, plagiarism detection, and identification of AI-generated content.

## Data Availability

The original contributions presented in the study are included in the article/supplementary material, further inquiries can be directed to the corresponding authors.

## References

[B1] Alsanoosy T. Shalbi B. Noor A. (2024). Authorship attribution for English short texts. Eng. Technol. Appl. Sci. Res. 14, 16419–16426. doi: 10.48084/etasr.8302

[B2] Berriche L. Larabi-Marie-Sainte S. (2024). Unveiling ChatGPT text using writing style. Heliyon 10:e32791. doi: 10.1016/j.heliyon.2024.e32976 38984302 PMC11231544

[B3] Bozza S. Roten C A. Jover A. Cammarota V. Pousaz L. Taroni F. (2023). A model-independent redundancy measure for human versus ChatGPT authorship discrimination using a Bayesian probabilistic approach. Sci. Rep. 13, 1–15. doi: 10.1038/s41598-023-46390-8 37932415 PMC10628141

[B4] Cumming A. (2001). Learning to write in a second language: two decades of research. Int. J. Engl. Stud. 1, 1–23.

[B5] Dugan L. Hwang A. Trhlik F. Ludan J. Zhu A. Xu H. . (2024). RAID: a shared benchmark for robust evaluation of machine-generated text detectors. arXiv [preprint]. *arXiv:2405.07940*. doi: 10.18653/v1/2024.acl-long.674

[B6] Ellis R. (2008). The Study of Second Language Acquisition, 2nd Ed. New York, NY: Oxford University Press.

[B7] Gritsai G. Voznyuk A. Grabovoy A. Chekhovich Y. (2024). Are AI detectors good enough? a survey on quality of datasets with machine-generated texts. arXiv [preprint]. *arXiv*:2410.14677.

[B8] He X. Lashkari A. H. Vombatkere N. Sharma D. P. (2024). Authorship attribution methods, challenges, and future research directions: a comprehensive survey. Information 15, 131–158. doi: 10.3390/info15030131

[B9] Huang B. Chen C. Shu K. (2024). Authorship attribution in the era of LLMs: problems, methodologies, and challenges. SIGKDD explorations 26, 21–43. doi: 10.1145/3715073.3715076 40276161 PMC12019761

[B10] Kumarage T. Liu H. (2023). Neural authorship attribution: stylometric analysis on large language models. arXiv [preprint]. *arXiv:2308.07305*. doi: 10.1109/CyberC58899.2023.00019

[B11] Liang W. Yuksekgonul M. Mao Y. Wu E. Zou J. (2023). GPT detectors are biased against non-native english writers. Patterns 4:100777. doi: 10.1016/j.patter.2023.100779 37521038 PMC10382961

[B12] Manchón R. M. (2011). “Situating the learning-to-write and writing-to-learn dimensions of L2 writing,” in Learning-to-Write and Writing-to-Learn in an Additional Language, ed. R. M. Manchón (Philadelphia, PA: John Benjamins Publishing Company), 3–14.

[B13] Mikolov T. Májovský M. Černý M Netuka D. (2024). Perfect detection of computer-generated text faces fundamental challenges. Cell Rep. Phys. Sci. 5:101433. doi: 10.1016/j.xcrp.2023.101769

[B14] Przystalski K. Argasiński J K. Grabska-Gradzińska I. Ochab J K. (2026). Stylometry recognizes human and LLM-generated texts in short samples. Exp. Syst. Appl. 296:129001. doi: 10.1016/j.eswa.2025.129001

[B15] Rahmanm S. M. M. Hasan M A. Islam M M. (2023). TDRLM: Stylometric learning for authorship verification by topic-debiasing. Expert Syst. Appl. 233:120745. doi: 10.1016/j.eswa.2023.120745

[B16] Selinker L. (1972). Interlanguage. Int. Rev. Appl. Linguist. Lang. Teach. 10, 209–232. doi: 10.1515/iral.1972.10.1-4.209

[B17] Tripto N I. Uchendu A. Le T Setzu M. Giannotti F. Lee D. (2023). HANSEN: Human and AI spoken text benchmark for authorship analysis. arXiv [preprint]. *arXiv:2310.16746*. doi: 10.18653/v1/2023.findings-emnlp.916

[B18] Zaitsu W. Jin M. (2023). Distinguishing ChatGPT-generated and human-written papers through Japanese stylometric analysis. arXiv [preprint]. *arXiv:2304.05534*. doi: 10.1371/journal.pone.0288453 PMC10411719 37556434

[B19] Zheng W. Jin M. (2023). A review on authorship attribution in text mining. WIREs Comput. Stat. 15:e1598. doi: 10.1002/wics.1584

